# Development of a severe influenza critical care curriculum and training materials for resource-limited settings

**DOI:** 10.1186/cc12437

**Published:** 2013-03-19

**Authors:** JV Diaz, P Lister, JR Ortiz, NK Adhikari, N Adhikari

**Affiliations:** 1 World Health Organization, San Francisco, CA, USA; 2 Great Ormond Street Hospital, London, UK; 3 University of Washington, Seattle, WA, USA; 4 Sunnybrook Health Sciences Centre and University of Toronto, Canada; 5 Health Sciences Centre and University of Toronto, Canada

## Introduction

The 2009 H1N1 pandemic caused surges of severely ill patients with viral pneumonia requiring ventilation and particularly affected high-dependency medical services such as ICUs in settings that lack sufficient personnel and resources to provide optimal care. Ministries of Health of several countries asked the World Health Organization (WHO) for clinical management guidance.

## Methods

We developed (2009 to 2011) and piloted (2011 to 2012) curriculum and training materials targeted at clinicians without formal critical care training who care for adult and paediatric patients with severe acute respiratory infections in ICUs in resource-limited settings.

## Results

With contributions from 37 global experts, we developed a 3-day course including 14 learning units in early recognition, patho-physiology, oxygen therapy, influenza diagnostics, infection control, resuscitation of septic shock, antimicrobial therapy, monitoring, lung-protective ventilation for acute respiratory distress syndrome, sedation, weaning, preventive care, quality improvement, and ethics. Teaching techniques are appropriate for adult learners and include short slideshow-based lectures, interactive small-group role-play sessions and a toolkit with practical resources such as checklists. With funding from the WHO and local Ministries of Health, the training course was piloted among 67 clinician participants in the Caribbean and Indonesia (29 and 38, respectively) and taught by external critical care specialists. Participants' daily evaluations rated all units as at least very good. Test scores to assess participant critical care knowledge improved significantly from before the training to immediately after (Caribbean, 58 to 80%; Indonesia, 56 to 75%; *P *0.001 for both).

## Conclusion

It was feasible to develop and deliver an advanced critical care curriculum and related training materials in a short, interactive workshop for noncritical care trained clinicians in resource-limited settings. However, the small-scale programme was labour and time intensive. Widespread dissemination of these materials requires identification of target countries, engagement of Ministries of Health, and secure funding. Longer-term evaluation is necessary to determine whether such programmes improve processes of care and clinical outcomes for critically ill patients.

